# Relationships between heritable dementia risk factors, cardiovascular risk factors in young adulthood, and midlife neuropsychological outcomes

**DOI:** 10.1177/13872877251401482

**Published:** 2026-01-06

**Authors:** Ivan Koychev, Nemanja Vaci, Justine Moonen, Graham Reid, Gregory Howgego, Kristine Yaffe, Ilya Nasrallah, Stephen Sidney, Christoph Jindra, John Gallacher, Lenore J. Launer

**Affiliations:** 1 Department of Psychiatry, University of Oxford, Oxford, UK; 2 Department of Psychology, University of Sheffield, Sheffield, UK; 3 Intramural Research Program, National Institute on Aging, National Institutes of Health, Baltimore, MD, USA; 4 Department of Experimental Psychology, University of Oxford, Oxford, UK; 5 Oxford Institute of Clinical Psychology Training and Research, University of Oxford, Oxford, UK; 6 Departments of Psychiatry, Neurology, and Epidemiology and Biostatistics, University of California, San Francisco, CA, USA; 7 Hospital of the University of Pennsylvania, Philadelphia, PA, USA; 8 Division of Research, Kaiser Permanente Northern California, Oakland, CA, USA; 9 Institute for Educational Quality Improvement (IQB), Humboldt Universität zu Berlin, Berlin, Germany

**Keywords:** Alzheimer's disease, *APOE4* carriership, dementia, dyslipidemia, modifiable risk factors

## Abstract

**Background:**

Selected cardiovascular factors, *APOE4* carriership, and family history (FH) are robust risk factors for Alzheimer's disease and dementia. While cardiovascular risk tends to affect cognition from midlife, it remains unclear whether heritable risk predicts cardiovascular health in young adulthood and midlife, and whether young-adult cardiovascular health predicts midlife cognition.

**Objective:**

We sought to examine how heritable dementia risk relates to cardiovascular health and how these cardiovascular risk factors in young adulthood predict midlife brain volumes and cognition.

**Methods:**

We used data from the CARDIA study, which followed 5115 individuals aged 18–30 at baseline over 30 years. Analyses focused on 2808 participants (Mean age = 60, *SD* = 3.58) who attended the 30-year visit. We examined associations between *APOE4* and FH with baseline and 30-year follow-up measures of cardiovascular risk factors (LDL-C, HDL-C, glucose, blood pressure, body mass index (BMI), smoking), cognition, and brain volumes.

**Results:**

*APOE4* carriers with FH had higher LDL-C and lower HDL-C levels as early as young adulthood, persisting into midlife. BMI and smoking were the only cardiovascular risk factors from young adulthood that predicted midlife cognition. There was no association between young adult cardiovascular risk factors and midlife brain volumes, but those with heritable dementia risk had larger brain volumes in regions vulnerable to midlife atrophy.

**Conclusions:**

*APOE4* carriership was associated with an unfavorable lipid profile that started in early adulthood and persisted to later life. Early cardiovascular risk was also associated with midlife cognition, which is earlier than studies typically focusing on later-life cognition.

## Introduction

Up to a third of all dementia cases are estimated to be due to preventable risk factors.^1^ Among these, midlife cardiovascular risk factors (CVRF) such as obesity, diabetes, hypertension, dyslipidemia, and smoking are some of the most robust predictors of cognitive decline and late-onset dementia.^1,2^ This effect is thought to be related to arterial changes and cerebral macro- and micro-vascular lesions, which affect blood supply to the brain's neurons.^3^ Improved CVRF treatment in the United States, as well as in European countries, over the past decades has been suggested as the main factor behind the slower-than-expected increase in dementia incidence.^4^ As such, tracking and understanding the optimal timing for intervening in modifiable risk factors associated with dementia, such as CVRF, is of clear importance.^5^ Despite the strong associations between mid-life CVRF and later-life dementia, it is unclear how heritable risk factors, such as family history of dementia and genetic carriership, as well as CVRF in young adulthood affect midlife cognition and brain health (before the onset of dementia or cognitive decline in later life). Recent studies have shown there to be a link between cardiovascular and immunological risk and cognition in young adulthood. For example, in one study, cardiovascular risk, as measured by CAIDE scores (Cardiovascular Risk Factors, Aging, and Incidence of Dementia) and immune system markers, such as IL-10, predicted lower cognitive scores in participants at age 28.^6^ In participants at 38 years old, total tau and mediators of the inflammatory response, such as IL-6 and IL-1β, also predicted reduced cognitive abilities in the same study. Dementia risk and cognitive abilities in younger people do remain less studied, however, and the longitudinal links between early risk factors and cognitive outcomes in midlife require further research. Understanding how heritable factors and CVRF from young adulthood associate with midlife brain-health and cognition may help guide earlier interventions before midlife in which the link between CVRF and later-life dementia has already been established.

Along with aging, *APOE4* carriership and family history of dementia are the chief non-modifiable risk factors for late-onset dementia. The apolipoprotein E protein plays a role in cholesterol metabolism and has been implicated in the clearance of cortical amyloid from the brain.^7^ It was first identified as a genetic risk factor for sporadic Alzheimer's disease (AD) in the early 1990s,^8^ a finding that has since been replicated in a large meta-analysis of genome-wide association studies.^9^ Population studies have estimated that homozygosity for *APOE4* increases the risk for all-cause dementia by a factor of six,^10^ while *APOE4* heterozygosity carriers a three times higher risk for AD.^11^ First-degree family history of dementia has also been associated with an increased risk for AD^12,13^ and has been accepted in genome-wide association studies as a proxy for AD/dementia risk.^14^ While having one or two first-degree relatives is associated with a 1.7- and 4.0-times higher risk for dementia,^13^ respectively, what is less clear in the literature is how these ‘non-modifiable’ risk factors for late-life dementia impact on cardiovascular health in young adulthood and whether cardiovascular risk seen in young-adulthood then associates with neurocognitive outcomes in midlife.

In the current paper, we examined how heritable risk factors for dementia impact on early cardiovascular health measures of cholesterol, blood glucose, body mass index, and blood pressure. We also sought to explore how these CVRF in young adulthood associate with midlife brain volumes and cognitive functioning. We hypothesized that family and genetic risk for dementia would be associated with poorer cardiovascular health measures. We also hypothesized that cardiovascular risk factors in young adulthood would be associated with reduced brain volumes and cognitive performance in midlife.

## Methods

### Study setting and participants

Data were taken from the Coronary Artery Risk Development in Young Adults Study (CARDIA), which investigated the development and determinants of clinical and subclinical cardiovascular disease and its risk factors. It recruited 5115 individuals aged 18–30 in 1985–1986 across four US Centers: Birmingham, AL; Chicago, IL; Minneapolis, MN; and Oakland, CA. The participants were invited back for assessments at Year 2, Year 5, Year 7, Year 10, Year 15, Year 20, Year 25, and Year 30. At the study visits, the participants underwent medical interviews and examinations and a variety of biomarkers relevant to cardiovascular health were recorded. Family history of dementia in first-degree relatives was recorded starting at Y30. *APOE* phenotype was determined from plasma samples collected during the Year 7 examination (1993–1994) by isoelectric focusing followed by immunoblotting as described elsewhere.^15^ Subsequently, people were classified into three distinct *APOE* groups: ε3 (*APOE33*), ε2 (*APOE22* and *APOE23*), and ε4 (*APOE34, APOE24*, and *APOE44*).

For the purposes of this study, we included data on demographics, family history of dementia (recorded at Y30), smoking (Y0, Y30), body mass index (Y0, Y30), blood pressure (Y0 and Y30), and plasma biomarkers, including Y0 and Y30 low density lipids (LDL-C), Y0 and Y30 high density lipids (HDL-C), and Y0 and Y30 fasting glucose levels. Tests of cognition, which are detailed below, were recorded at Y30, and magnetic resonance imaging (MRI) assessments were taken at Y25 and Y30.

### MRI methods

MRI data were collected on a subset of participants as a part of the CARDIA brain MRI sub-study, described elsewhere with details on the imaging acquisition, processing protocols and quality control.^16^ Briefly, the CARDIA MRI acquisition protocol included: (i) Sagittal T1-weighted 3D magnetization-prepared rapid gradient echo (MPRAGE) data acquired with TR/TE/TI = 1900/2.9/900 ms, flip angle = 90, bandwidth = 170 Hz/pixel, voxel size = 1 × 1 × 1 mm, matrix = 256 × 256, slices = 176, (ii) Sagittal T2 weighted MRI were obtained with TR/TE = 3200/409 ms, bandwidth = 750 Hz/pixel, voxel size = 1 × 1 × 1 mm, matrix = 258 × 256, slices = 176, and iii) Sagittal Fluid Attenuated Inversion Recovery (FLAIR) MRI were acquired with TR/TE/TI = 6000/160/2200 ms, bandwidth = 930 Hz/px, voxel size = 1 × 1 × 1 mm, matrix = 258 × 221, slices = 160.

MRI data were transferred to a central archive and processed at the CARDIA MRI Reading Center at the University of Pennsylvania using an automated pipeline. Images with incidental findings, motion artifacts, or poor image quality affecting image processing were excluded from analyses. To estimate brain volumes, we used a multi-atlas label fusion method (MUSE), which has been previously described.^17^ We then added together the volumes of grey matter ROIs (medial frontal, frontal insular, thalamus, limbic cingulate, limbic medial temporal, lateral temporal, parietal lateral, and parietal medial grey matter) that correspond to the regions included in a validated index which captures patterns of brain aging atrophy (Spatial Pattern of Atrophy for Recognition of Brain Ageing, SPARE BA).^18,19^

### Cognition

Standardized tests, including the Stroop test,^20^ Digit Symbol Substitution Test (DSST),^21^ and Montreal Cognitive Assessment (MoCA),^22^ were administered at Year 30 to evaluate multiple domains of cognition. The Stroop test evaluates executive function, including the ability to view complex visual stimuli and respond to 1 stimulus dimension while suppressing the response to another dimension. The test is scored by seconds taken to spell color words printed in a different color ink, plus the number of errors. Stroop scores range from 1 to 160, and higher seconds plus errors score indicate worse performance. We assessed psychomotor speed with the DSST (score range 0 to 133) in which having more correct digits indicates better performance. MoCA is a test of global cognition that evaluates multiple cognitive domains with a range of 0 to 30 in which higher scores indicate overall better cognitive abilities.^22^ For more details on the full protocol for cognitive testing, see previously published literature.^23^

### Cardiovascular risk factors

Body mass index (BMI) was calculated as participants’ weight in kilograms divided by their height in meters square. Blood pressure was measured from participants’ right arm after 5 min of rest using a random-zero sphygmomanometer to measure 3 systolic and fifth-phase diastolic blood pressure every 60 s. An average of the 2^nd^ and 3^rd^ measures was then calculated for each participant to be used in the analyses. Levels of fasting glucose were measured by Linco Research, Inc, using a hexokinase ultraviolet method. Total cholesterol and high-density lipoprotein cholesterol (HDL-C) were measured enzymatically by the Northwest Lipid Laboratory. The Friedewald equation was used to calculate low-density lipoprotein cholesterol (LDL-C).^24^ Age,^25^ sex,^26^ race,^27^ education,^28^ and smoking status^29^ were used as covariates as they are known to predict the cardiovascular risk factors used as predictors in our analyses.

### Analytical sample

After excluding the participants that did not have information on *APOE4* status and family history of dementia, our study included measurements from 2808 participants. For the cognitive function analyses, there were 2506 out of 2808 participants after listwise deleting participants with missing observations (153 observations) and extreme values in the tails of the cardiovascular measures (149 observations). Outliers were removed since coefficient-level p values in path analyses (or structural equation modelling more generally) are sensitive to departures form multivariate normality and extreme (leverage) values. As such, we restricted tails as we felt it appropriate to conduct conservative tests that, while perhaps inflating beta error, would control the type I (alpha) error rate. MRI metrics were acquired on 714 individuals. For these analyses we used the MRI measurement from Y30 or Y25 if there was only a single MRI measure. If both measurements were present, we averaged the two measures.

### Statistical analyses

We used structural equation modelling (with bootstrapping) to investigate the effects of *APOE4* and family history on a range of cardiovascular factors, brain volumetric measures, and cognitive performance. By including direct and indirect paths within the multivariate models, we were able to assess the indirect effects of cardiovascular risk factors in young adulthood on the relationship between heritable dementia risk factors (i.e., the predictor) and brain health (i.e., the outcomes).To test the joint and independent effects of genetic susceptibility and CVD risk factors, we created four groups: (i) *APOE4* negative and family history negative (A-F-), (ii) *APOE4* positive and family history negative (A + F-), (iii) *APOE4* negative and family history positive (A-F+), and finally, (iv) *APOE4* positive and family history positive (A + F+). The models utilized standard dummy coding of categorical variables where one level (A-F- in our case) was excluded from the estimation and was used as a reference level. All other levels were then compared with the reference level and model estimates reflected differences between factor levels. We ran sensitivity analyses which excluded previously imputed missing data points and persons in the tails of the cardio-vascular RFs (see online supplemental materials: Sensitivity analysis 1 and 2). Results did not substantially differ. We used two measures to evaluate the overall model fit: the Comparative fit index (CFI) and root mean square error of approximation (RMSEA). CFI is an incremental fit of the model measure. It calculates how well the proposed model explains the data in comparison to the null (only intercept) model (with values above 0.95 indicating an acceptable model fit). RMSEA is an absolute fit index as it calculates how far the proposed model is from the perfect fit to the observed values (values below < 0.05 indicate an acceptable model fit). These acceptability estimates are based on a comparison between the model implied covariance matrix and the observed covariance matrix.

### Cardiovascular model

The initial model estimated the association of *APOE4* and family history with cardiovascular risk. In addition to these heritable factors, we controlled for the effects of smoking (smoker, ex-smoker, non-smoker), age, education, sex and race of participants, and the study site. Outcomes included Y0 and Y30 measures of low-density lipoprotein cholesterol (LDL-C mmol/L) and high-density lipoprotein cholesterol (HDL-C mmol/L), plasma glucose (mg/dL), body mass index (BMI), and systolic and diastolic blood pressure (SBP and DBP mmHg, respectively). We controlled for the covariances between the cardiovascular measurements taken at the same time point (e.g., LDL-C at Y30 with HDL-C at Y30). Additionally, we used measures of modification indices to calculate which covariance pathways considerably improved the fit of the model.^30^ Finally, for the Y30 analysis we included autoregressive terms in the analysis, whereby the Y30 measurement was regressed onto the Y0 measure. For the complete model specification, please see the online supplementary materials. The CFI and RMSEA model fit estimates were 0.997 and 0.017 (95% CI: 0.010–0.023), both indicating the model was an acceptable fit to the data.

### Cognitive model

We used path analyses to investigate both the direct effects of heritability factors (*APOE4* and family history) and cardiovascular risk factors on cognition (i.e., MoCA, DSST, and Stroop test), as well as the indirect effects of cardiovascular risk factors where direct effects were detected between the heritable factors and cognitive outcomes. The cardiovascular variables (LDL-C, HDL-C, glucose, BMI, systolic, and diastolic blood pressure measured at Y0) were regressed onto *APOE4* and family history status, while controlling for age, race, sex, smoking status, education, and study site. In addition, we modelled measures of cognitive performance as a function of cardiovascular measures at Y0, *APOE4*, and family history of dementia and all controls (see the full model specification in the cognition analyses section in the Supplemental Material). We included a full covariance structure between the dependent variables in the model and z-transformed all continuous variables. The overall performance of the model indicated good fit to the data, with CFI reaching 0.996 and RMSEA 0.026 (95% CI: 0.017–0.035).

### SPARE BA model

Like the cognitive data, the analysis of the neuroimaging data also utilized path modelling. This approach allowed the quantification of effects that heritability factors (*APOE4* and family history) and cardiovascular risk factors had on SPARE BA regions by estimating their direct influence and indirect influence through cardiovascular measures. The cardiovascular outcomes included Y0 measurements for all cardiovascular measures. *APOE4* carriership, family history, age, race, sex, smoking status, and study site were included as predictors of the dependent outcome. We included a full covariance structure between the dependent variables in the model. Brain volumes were normalized to total intracranial volume and z-transformed. The overall performance of the spare BA model showed poor fit to the data, with CFI score of 0.56 and RMSEA of 0.180.

## Results

### Participants

A total of 2808 participants were included in the analyses. A summary table of the number of participants with genetic risk (i.e., carrying at least one ε4 allele) and family risk can be seen in Table 1. A summary of participant data across risk factors, demographics, and control variables can be seen in Table 2. The distribution of participants was as follows: A-F- = 1514; A + F- = 636; A-F + = 436; A + F + =222.

**Table 1.** A summary table of the proportion of participants with genetic and family risk.

		Family History		
		No	Yes	**Total**
*APOE4*	No	1514	436	1950
	Yes	636	658	858
	**Total**	2150	658	2808

**Table 2.** Descriptive statistics of the study sample.

	Total	A−F−	A + F−	A−F+	A + F+
Age (years)					
Year 0	2808	24.95	24.68	26.07	25.68
Year 30	2808	55.03	54.75	56.16	55.81
Race (% non-white)	2808	41%	59%	33%	44%
Never smoker (%)					
Year 0	2792	61%	63%	61%	63%
Year 30	2780	63%	65%	64%	62%
Education	2796	15.28 (2.59)	14.80 (2.57)	15.94 (2.67)	15.34 (2.57)
LDL-C (mmol/L)					
Year 0	2782	106.31 (30.34)	115.70 (30.09)	106.45 (29.21)	116.13 (29.11)
Year 30	2758	107.42 (32.89)	114.91 (32.14)	109.55 (30.43)	118.48 (33.30)
HDL-C (mmol/L)					
Year 0	2789	54.23 (12.91)	52.18 (12.30)	54.41 (12.64)	51.90 (12.53)
Year 30	2783	60.42 (19.27)	57.17 (16.88)	61.32 (18.00)	58.76 (17.28)
Glucose (mg/dL)					
Year 0	2772	81.73 (9.57)	81.66 (8.66)	81.74 (8.02)	81.71 (9.39)
Year 30	2777	102.66 (32.18)	102.41 (27.94)	103.42 (33.50)	100.67 (26.96)
BMI					
Year 0	2800	24.22 (4.65)	24.74 (4.82)	24.24 (4.69)	24.33 (4.92)
Year 30	2803	30.41 (7.29)	31.21 (7.05)	29.62 (6.96)	29.88 (6.67)
Systolic BP (mmHg)					
Year 0	2808	110.24 (10.54)	110.43 (10.41)	109.19 (10.96)	107.60 (10.41)
Year 30	2808	120.16 (16.61)	120.45 (15.14)	116.77 (15.09)	117.49 (14.12)
Diastolic BP (mmHg)					
Year 0	2808	68.65 (9.30)	68.49 (9.28)	68.82 (8.69)	67.69 (9.54)
Year 30	2808	73.66 (11.03)	73.05 (10.72)	70.48 (9.84)	72.31 (10.25)
MoCA	2649	23.97 (3.96)	23.33 (3.89)	24.75 (3.40)	24.21 (3.73)
DSST	2664	68.32 (16.95)	66.03 (16.76)	70.15 (16.80)	68.89 (14.96)
STROOP	2627	22.47 (11.51)	24.04 (11.92)	21.70 (10.54)	21.67 (9.70)
SPARE-BA	750	0.191 (0.010)	0.192 (0.012)	0.193 (0.011)	0.195 (0.012)

### Cardiovascular model

#### Low-density lipoprotein cholesterol

Results showed a significant effect of *APOE4* carriership on LDL-C at both baseline and follow-up. Participants with *APOE4* carriership and no family history status (A + F-) had higher levels of LDL-C at both baseline and 30-year follow-up (Y0: β = 8.72, SE = 1.48, z = 5.88, p < 0.001 and Y30: β = 8.50, SE = 2.19, z = 3.88, p < 0.001) in comparison to the reference group (non-carrier and no family history, A-F-). The same relationship was observed for those with both heritability factors, A + F+, relative to the A-F- controls (Y0: β = 9.25, SE = 2.21, z = 4.18, p < 0.001 and Y30: β = 11.60, SE = 2.85, z = 3.88, p < 0.001).

#### High-density lipoprotein cholesterol

Results for HDL-C showed the opposite relationship to LDL-C above in the association between *APOE4* carriership and HDL-C. Participants with *APOE4* carriership, either with or without family history status, had lower levels at Y0 relative to the A-F- group (A + F-: β = −2.06, SE = 0.59, z = −3.46, p < 0.01 and A + F+: β = −2.60, SE = 0.89, z = −2.91, p < 0.01), with no difference for A-F + group (A + F-: β = 0.212, SE = 0.701, z = 0.30, p > 0.05). At Y30, there was no evidence of a statistically significant difference in HDL-C for the A + F- group (β = −1.18, SE = 0.67, z = −1.77, p > 0.05), A-F + group (β = −0.21, SE = 0.79, z = −0.27, p > 0.05), or the A + F + group (β = −0.02, SE = 1.08, z = −0.02, p > 0.05) relative to the A-F- control group.

#### Plasma glucose

Results revealed no significant effect of *APOE4* carriership on plasma glucose levels at baseline or at 30-year follow-up. At baseline, neither the A + F- group (β = 0.16, SE = 0.35, z = 0.46, p > 0.05) nor the A + F + group (β = 0.11, SE = 0.68, z = 0.16, p > 0.05) had statistically different levels of plasma glucose compared to the A-F- comparison group. The same pattern of results was found at the 30-year follow-up for both the A + F- group (β = 0.07, SE = 0.92, z = 0.08, p > 0.05) and the A + F + group (β = −1.60, SE = 1.41, z = −1.14, p > 0.05). Note that there was a significant effect of time on plasma glucose with measures being higher at the 30-year follow-up compared to baseline with a mean difference of 20.87, t(2742) = 35.00, p < 0.001.

#### Body mass index

Results revealed no significant effect of *APOE4* carriership on body mass index at baseline or at 30-year follow-up. At baseline, neither the A + F- group (β = 0.16, SE = 0.23, z = 0.72, p > 0.05) nor the A + F + group (β = −0.03, SE = 0.36, z = −0.09, p > 0.05) had statistically different body mass indexes compared to the A-F- comparison group. The same pattern of results was found at the 30-year follow-up for both the A + F- group (β = −0.02, SE = 0.26, z = −0.06, p > 0.05) and the A + F + group (β = −0.53, SE = 0.38, z = −1.38, p > 0.05). Note that there was a significant effect of time on body mass index with measures being higher at the 30-year follow-up compared to baseline with a mean difference of 6.09, t(2794) = 58.43, p < 0.001.

#### Blood pressure

Blood pressure was associated with *APOE4* and family history status, where APOE4 carriers with family history had lower levels of systolic blood pressure at Y0 relative to the A-F- group (A + F+: β = −2.289, SE = 0.704, z = −3.252, p < 0.01), while in Y30 we observed this difference only in the case of non *APOE4* carriers with family history (A-F+; β = −2.345, SE = 0.815, z = −2.877, p < 0.01). Results on diastolic blood pressure showed parallel results, where *APOE4* carriers with family history had lower levels at Y0 relative to the A-F- group (β = −1.504, SE = 0.703, z = −2.139, p < 0.05), while non *APOE4* carriers with family history had lower levels at Y30 relative to the A-F- group (β = −2.373, SE = 0.546, z = −4.344, p < 0.01).

#### Cognition model

There were no significant effects of *APOE4* or family history on cognitive performance (Table 3). Furthermore, although *APOE4* was associated with increased levels of LDL-C and decreased HDL-C levels, as well as changes in blood pressure, these cardiovascular risk factors at Y0 were not associated with cognitive performance at Y30 either. Only body mass index measured at baseline was associated with scores on the Stroop test and MoCA at Y30. And lastly, only smokers at Y0 compared to non-smokers had lower MoCA scores at Y30 (β = −0.14, SE = 0.06, z = −2.33, p < 0.05). As there was no association between predictor (genetic risk and family history of dementia) and outcome (cognition), formal tests of mediation effects through cardiovascular risk factors are not reported.

**Table 3.** Main effects of family history, genetic risk, and cardiovascular factors on cognitive outcomes.

	β	SE	Z	p
Stroop				
A + F−	0.07	0.07	1.07	0.28
A−F+	0.08	0.06	1.16	0.24
A + F+	0.09	0.07	1.22	0.22
LDL−C (mmol/L)	−0.01	0.02	−0.48	0.63
HDL−C (mmol/L)	−0.02	0.02	−1.00	0.32
Plasma Glucose (mg/dL)	−0.01	0.02	−0.42	0.68
Body Mass Index	0.05	0.02	2.08	0.04
Systolic Blood Pressure (mmHg)	−0.001	0.02	−0.05	0.96
Diastolic Blood Pressure (mmHg)	0.03	0.02	1.09	0.28
Digital Symbol Substitution Test				
A + F−	0.02	0.07	0.27	0.79
A−F+	0.02	0.07	0.35	0.72
A + F+	0.02	0.08	0.27	0.78
LDL−C (mmol/L)	0.002	0.02	0.09	0.93
HDL−C (mmol/L)	−0.01	0.02	−0.48	0.63
Plasma Glucose (mg/dL)	0.002	0.02	0.12	0.90
Body Mass Index	−0.03	0.02	−1.79	0.07
Systolic Blood Pressure (mmHg)	−0.02	0.02	−0.61	0.54
Diastolic Blood Pressure (mmHg)	−0.002	0.02	−0.09	0.93
Montreal Cognitive Assessment				
A + F−	−0.06	0.06	−1.00	0.32
A−F+	−0.05	0.06	−0.84	0.39
A + F+	0.01	0.07	0.21	0.84
LDL−C (mmol/L)	0.01	0.02	0.62	0.54
HDL−C (mmol/L)	−0.01	0.02	−0.32	0.75
Plasma Glucose (mg/dL)	0.01	0.02	0.69	0.49
Body Mass Index	−0.06	0.02	−3.37	0.001
Systolic Blood Pressure (mmHg)	−0.001	0.02	−0.03	0.98
Diastolic Blood Pressure (mmHg)	−0.2	0.02	−1.10	0.27

### SPARE-BA model

Results suggested that people with a family history of dementia and positive *APOE* status had increased volumes of SPARE-BA region (Table 4). The risk factors and cardiovascular risk factors had no significant main effect on SPARE-BA. As such, formal tests of mediation effects through cardiovascular risk factors are not reported.

**Table 4.** Main effects of family history, genetic risk, and cardiovascular factors on brain outcomes.

	β	SE	Z	p
A + F-	0.02	0.09	0.19	0.85
A-F+	0.12	0.10	1.26	0.20
A + F+	0.33	0.13	2.54	0.01
LDL-C	−0.05	0.04	−1.41	0.16
HDL-C	−0.04	0.04	−1.18	0.24
Plasma Glucose	−0.04	0.04	−1.32	0.19
Body Mass Index	0.005	0.04	0.13	0.90
Systolic Blood Pressure	0.03	0.04	0.78	0.44
Diastolic Blood Pressure	−0.02	0.04	−0.41	0.68

## Discussion

The current study sought to investigate how heritable risk factors for dementia impact on cardiovascular health in young adulthood and how CVRF in young adulthood associate with brain volumes and cognitive function in midlife. We found that *APOE4* carriership and family history of dementia associated with worse lipid profiles (higher LDL-C and lower HDL-C) starting from early adulthood and extending to midlife 30 years later. While there were no associations between heritable risk factors and cognitive abilities in midlife, there was some evidence that CVRF in young adulthood associated with poorer cognition in midlife. Specifically, we found that higher body mass index in young adulthood was associated with poorer performance on the Stroop test (impaired executive function in the domain of inhibition) and poorer overall cognition, as measured by the MoCA. We also found that smoking in young adulthood was associated with poorer overall cognition in midlife. As for volumetric measures of brain structure, we found that people with heritable dementia risk factors (i.e., familiar history and *APOE4* carriership) had larger brain volumes in regions generally seen to show age-related atrophy.

*APOE4* allele carriership is the strongest genetic susceptibility risk factor for late-onset dementia.^31^ However, the exact mechanisms by which it exerts this effect are likely complex and are modified by interactions with other genes and the environment. That *APOE4* should relate to LDL-C levels is not surprising based on current knowledge of lipid turnover. The ApoE protein is known to regulate the clearance of lipoproteins through their binding to cell-surface lipoprotein receptors and that this affinity has been shown to be higher for ε4 relative to ε3 or ε2 carriers.^32^ However, how and when lipid dysregulation exacerbates the risk of dementia is still unclear.^33^ Many mechanisms have been proposed, including increasing pathogenic Aβ production,^34–38^ microglial dysfunction and inflammation,^35,36,38^ oxidative stress,^35^ endolysosomal dysfunction,^36^ tau pathology,^37–39^ and the production of cytotoxic oxysterols as a result of cholesterol catabolism.^37^

Perhaps the most striking finding of this study was how early in the life course *APOE4* was associated with cardiovascular factors, which have been linked to dementia risk in prior research. It has previously been demonstrated that *APOE* carriership exerts an effect on lipid profiles in middle age, with the Framingham Heart Study cohort showing elevated LDL-C cholesterol levels in *APOE4* carriers in a sample of nearly 600 participants.^40^ The data presented here expands on that finding, demonstrating in a much larger and more diverse sample that the abnormal lipid profile associated with *APOE4* begins early in adulthood and is sustained over a period of at least 30 years. It has been demonstrated that for coronary heart disease, significantly greater risk reduction is achieved with early lipid-lowering therapy (LLT). As such, it is possible that a similar relationship between duration of uncorrected lipid abnormality and dementia may exist, but available data are inconclusive at this point.^41–44^ Population studies have shown that LLT is effective in mitigating *APOE4* associated risk independent of cholesterol levels,^45^ suggesting a tentative relationship between LLT use and dementia risk in the context of limited evidence. Review papers and mendelian randomization studies, however, do not generally support a relationship between statin treatment and cognition, questioning the benefits of such interventions on dementia risk as suggested in population studies.^43,44^ There has been a trend towards earlier initiation of statin treatment^42^ with the 10,000 patient Eliminate Coronary Artery Disease (ECAD) trial looking at statin use over 10 years in patients as young as 35.^46^ Follow-up of the cognitive outcomes of participants in such trials may yield particularly valuable information about the preventative potential of LLT in the context of the *APOE* genotype. Some research has suggested that lipid levels of *APOE4* carriers are most susceptible to dietary changes and so lifestyle modification may also represent an avenue of dementia risk reduction, notwithstanding the inconclusiveness of the available evidence concerning the use of LLT pharmaceuticals.^47^ As the practice of medicine becomes more personalized, understanding the interaction between cardiovascular risk factors and genotype may lay the ground for targeted programs to reduce future risk of dementia starting much earlier in the lifespan.

Indeed, our finding of an abnormal lipid profile in *APOE4* carriers is in line with a body of research demonstrating significant links between cardiovascular health and dementia. Poor cardiovascular health contributes to dementia both directly, through cerebrovascular pathology causing a degree of vascular dementia^48,49^ and impaired regulation of cerebral blood flow,^50^ and indirectly by accelerating the development of Alzheimer's disease.^48^The evidence for a strong cardiovascular risk component in AD pathogenesis is further substantiated by longitudinal studies consistently reporting that poorer cardiovascular profile associates with future risk for dementia.^51,52^ Of the CVRF assessed in this study, we found that BMI in young adulthood associated with poorer cognitive abilities in midlife, which may be an early indicator of dementia-related cognitive decline. While existing research has demonstrated that midlife BMI associates with later-life cognitive impairment and dementia risk, we showed that these effects may be detectable much earlier in the life course. In previous research it has been shown that obesity in early adulthood is associated with increased odds for dementia in late adulthood. But here we show that higher BMI in early adulthood is associated with changes in cognition much earlier than a dementia diagnosis, which is usually made in the 8^th^ decade of life. In line with other studies, we also found that BMI increased over time in our sample, which is typically reported with ageing due to changes in body composition and changes in physical activity and metabolism across the lifespan.^53^ Similarly, we also found that fasting glucose increased over time, which fits well with existing research, which tends to show progressive declines in insulin sensitivity and compensatory hyperinsulinemia as people age.^54,55^ We therefore note that these trends likely contributed to the impaired fasting glucose and elevated BMI that we observed at year 30 in our sample.

These findings are significant as, in the absence of a cure for dementia, there has been increasing focus on the role of intervening in modifiable risk factors to reduce dementia burden earlier in the lifespan.^56,57^ The progression of dementia is generally split into three stages from the preclinical phase to mild cognitive impairment and lastly to dementia. While general screening for dementia risk is generally not advised, knowing which CVRF present elevated odds for cognitive impairment can help guide advice from primary care givers on the control of modifiable risk factors and other chronic conditions. Research has shown that behavioral change is easier and longer lasting earlier in the lifespan and so knowledge that obesity carries risk for cognitive decline in midlife (and not only later life) is important for timely and efficacious risk reduction. Indeed, early detection of mild cognitive impairment is one approach in successfully tackling the personal and social burden of dementia. Primary caregivers lack the infrastructure to easily detect those with beginning cognitive impairment and so knowledge of the CVRF that may carry highest risk for the earliest changes in cognition in the lifespan is indispensable. In a survey of primary caregivers, it was suggested that there is a gap in their knowledge on which cognitive assessments to use in measuring early changes in cognition. The results of this study suggest that assessing executive function (i.e., inhibition) and general cognitive abilities in people with an early history of smoking and obesity may be useful in detecting the earliest of changes in cognitive abilities that may be apparent before mild cognitive impairment as early as midlife.

However, there are a few limitations in the present study that require mentioning. First, contrary to our initial hypothesis, our analyses did not reveal any significant associations between CVRF during young adulthood and brain volumes in midlife within the brain regions that are conventionally linked with cortical aging. This could be attributed to the selection criteria of our participant sample from the CARDIA study, which focused on individuals with varying genetic predispositions for dementia. It is plausible that those with a higher genetic risk demonstrated a degree of cognitive resilience owing to mechanisms related to brain reserve. This idea is supported by the finding that participants with genetic risk and a family history of dementia exhibited larger brain volumes compared to those without these genetic and familial risk factors. Indeed, the potential for collider bias might have influenced our findings by concealing the impact of CVRF on cognition and brain structure, assuming individuals with higher heritable dementia risk exhibited greater brain reserve, manifesting as larger brain volumes. Secondly, it is also conceivable that the relationships between specific CVRF, brain health, cognitive function, and heritable dementia risk might only emerge in later midlife stages or even in the late-life phase. Consequently, the age distribution of our sample might have masked potential mediation effects among these variables, as well as the direct effects between some CVRF in young adulthood and some cognitive and brain-related outcomes in midlife. Relatedly, just over half of the original sample were included in the analyses at the 30-year follow-up, representing sizeable attrition, which may have influenced the results of the current study. And lastly, cognition was only sampled once at the 30-year follow-up, representing another limitation of the study.

Nevertheless, this study has contributed valuable insights to our understanding of the connections between heritable dementia risk factors, CVRF, and neuropsychological functioning from early adulthood to midlife. Our findings highlight the association between *APOE4* carriership and a family history of dementia, and unfavorable lipid profiles in early adulthood. We also observed links between obesity and smoking during early adulthood and compromised cognition in midlife much earlier than previous research focusing on later life cognition. These findings contribute to our understanding of the role of CVRF in young adulthood and cognition 30 years later.
